# Nanoscale *π*–*π* stacked molecules are bound by collective charge fluctuations

**DOI:** 10.1038/ncomms14052

**Published:** 2017-02-07

**Authors:** Jan Hermann, Dario Alfè, Alexandre Tkatchenko

**Affiliations:** 1 Fritz-Haber-Institut der Max-Planck-Gesellschaft, Faradayweg 4–6, 14195 Berlin, Germany; 2 Department of Earth Sciences, University College London, London WC1E 6BT, UK; 3 Department of Physics and Astronomy, University College London, London WC1E 6BT, UK; 4 London Centre for Nanotechnology and Thomas Young Centre@UCL, University College London, London WC1E 6BT, UK; 5 Physics and Materials Science Research Unit, University of Luxembourg, 162a Avenue de la Faiencerie, Luxembourg L-1511, Luxembourg

## Abstract

Non-covalent *π*−*π* interactions are central to chemical and biological processes, yet the full understanding of their origin that would unite the simplicity of empirical approaches with the accuracy of quantum calculations is still missing. Here we employ a quantum-mechanical Hamiltonian model for van der Waals interactions, to demonstrate that intermolecular electron correlation in large supramolecular complexes at equilibrium distances is appropriately described by collective charge fluctuations. We visualize these fluctuations and provide connections both to orbital-based approaches to electron correlation, as well as to the simple London pairwise picture. The reported binding energies of ten supramolecular complexes obtained from the quantum-mechanical fluctuation model joined with density functional calculations are within 5% of the reference energies calculated with the diffusion quantum Monte-Carlo method. Our analysis suggests that *π*−*π* stacking in supramolecular complexes can be characterized by strong contributions to the binding energy from delocalized, collective charge fluctuations—in contrast to complexes with other types of bonding.

## Abstract (web summary)

Attractive, non-covalent interactions between aromatic rings—termed *π*−*π* stacking—is common in chemistry but difficult to model. Here the authors report a quantum-mechanical model to show the importance of collective charge fluctuations for understanding pi-stacked supramolecular systems.

The non-covalent *π*−*π* interactions between conjugated aromatic rings play a key role in a wide range of chemical and biological processes. These interactions contribute significantly to nucleobase stacking in RNA and DNA^1^, protein folding^2^, molecular recognition^3^, template-directed synthesis^4^ and assembly of van der Waals (vdW) heterostructures^5^. Despite intense experimental and theoretical studies, the conceptual understanding of *π*−*π* interactions in these complex systems is still largely based on small model systems such as the prototypical benzene dimer. Hunter and Sanders^6^ coined a classical perspective on *π*−*π* interactions, in which they emphasized the electrostatic quadrupole interactions between *π* orbitals. With later advances in electronic structure theory, it became clear that the reality is in fact an intricate interplay between electrostatic interactions, Pauli repulsion and London dispersion. Countless efforts have been put into the correct prediction of the most stable conformer of the benzene dimer, resulting in the excellent accuracy of ∼0.1 kcal mol^−1^ (refs 7, 8, 9, 10, 11, 12). However, due to the complex mathematical structure of high-level quantum chemistry methods, the complete understanding of the nature of the binding remains a challenge even in such relatively small systems. This situation even led to recent suggestions that there is really nothing special about *π*−*π* stacking in terms of intermolecular interactions, and that the term should be abandoned altogether^13^,^14^. On the other hand, Dobson and others^15^ have emphasized that collective plasmon fluctuations in zero-gap one-dimensional and two-dimensional systems, including conjugated graphene sheets, can lead to unusual power laws for the binding energies at separations beyond 10 nm^15^,^16^. The transition from the zero to finite gap between the highest occupied and lowest unoccupied molecular orbital (HOMO-LUMO) was further investigated by Misquitta *et al*.^17^ who related the difference to the inherently non-local response of the zero-gap systems. The plasmon-based approaches are effective for revealing asymptotic behaviour of vdW interactions between prototypical low-dimensional systems. However, conceptual understanding of interactions for equilibrium molecular geometries and their quantitative description remains an open problem.

In this work, we present a viewpoint on binding in supramolecular *π*−*π* systems that is based on correlation of collective quantum electron-density fluctuations, while being backed up by a quantitative model that is able to provide highly accurate predictions of binding energies. To this end, we employ the many-body dispersion (MBD) method^18^,^19^, which captures the anisotropy and collective nature of long-range correlation in such systems, while maintaining a wavefunction that is transparent enough for further analysis. We establish validity of this model by comparing with the binding energies of ten supramolecular complexes obtained by high-level diffusion quantum Monte-Carlo (DQMC) calculations. We then proceed to show how the correlation mechanism in MBD corresponds to that of virtual electronic excitations to unoccupied *π*-like orbitals in correlated quantum chemistry methods. This enables us to interpret the fluctuations in this model directly as correlated charge oscillations of the electronic clouds. We provide visual representation of these collective fluctuations and show how they build on, extend and generalize the atomic pairwise picture of London dispersion from small model molecules to large realistic systems. Furthermore, we decompose the total binding energies of various types of supramolecular complexes into the individual fluctuation modes and thus provide evidence that the collective nature of these fluctuations is characteristic of the conjugated systems.

## Results

### Binding energies of three supramolecular complexes

**Figure 1:**
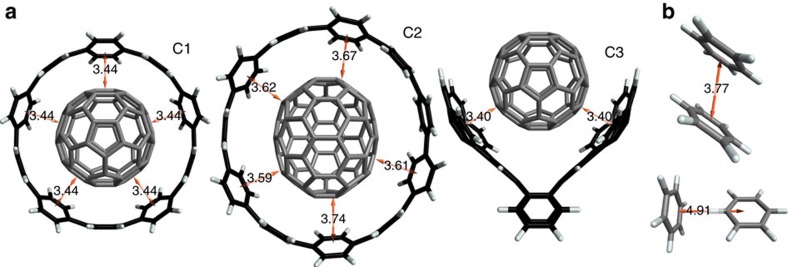
Equilibrium structures of studied *π*−*π* stacked complexes. Marked distances are in ångströms. Geometries of all structures are available as XYZ files in Supplementary Data 1. (**a**) Three supramolecular complexes comprising the fullerene C70 molecule (grey) as a guest in three different hosts (black and white): [10]-and [11]CPP, and a buckycatcher molecule, labelled C1, C2 and C3, respectively. (**b**) Benzene dimer in its two nearly degenerate conformations: stacked parallel-displaced (top) and T-shaped (bottom).

**Figure 2:**
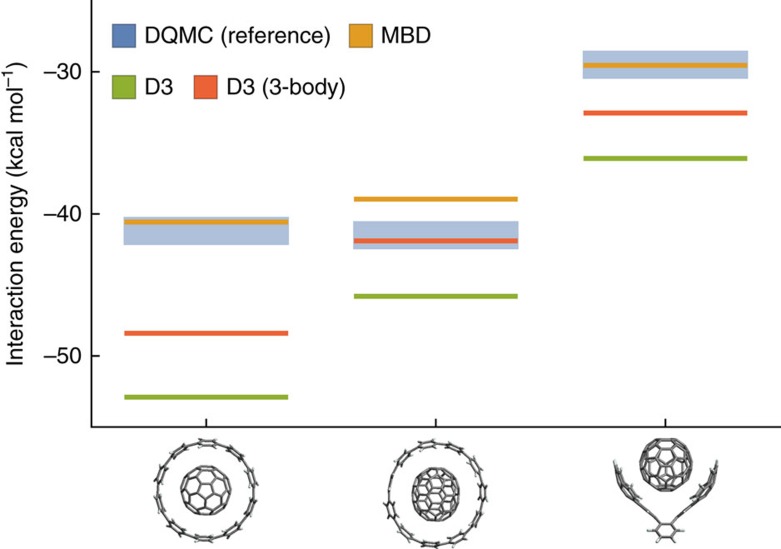
Interaction energies of complexes C1–C3 (kcal mol^−1^). The energies are evaluated with respect to relaxed fragments (see Supplementary Data 1). DQMC is the reference diffusion quantum Monte-Carlo method^31^. The blue bar indicates the statistical sampling errors in the energy, which are inherent to the method. MBD is the MBD method^19^ calculated on top of the PBE exchange-correlation density functional^58^. D3 is the DFT-D3 approach^59^ with the optional three-body correction on top of the PW6B95 density functional; the values were taken from refs 23, 24. Numerical values are presented in Supplementary Table 1.

We start by analysing a set of three already synthesized^20^,^21^ and theoretically investigated^22^,^23^,^24^ supramolecular *π*−*π* complexes (Fig. 1a), consisting of a C70 fullerene guest molecule hosted by two different cycloparaphenylenes (CPPs; C1 and C2) and a buckycatcher molecule (C3). The CPPs are the simplest structural units of ‘armchair' carbon nanotubes and their complexes are precursors of fullerene peapods^25^,^26^. The buckycatcher complex, on the other hand, represents a class of convex–concave *π*−*π* systems^27^. The experimental free energies of association of these complexes are 7, 7 (ref. 28) and 5 kcal mol^−1^ (ref. 29), respectively, making C1 and C2 degenerate in terms of stability. Reliable theoretical prediction of free energies of association is yet an unsolved task burdened by numerous complications, especially concerning the contribution of solvation effects^30^. Our focus here is instead on the electronic origin of *π*−*π* bonding and hence the relevant quantities are the binding energies. As we need to validate our vdW model as described below, we require reliable reference results to begin with. To avoid the issues associated with estimating binding energies from experimental free energies, we have employed a theoretical reference in the form of DQMC binding energies as an alternative. DQMC approximates the exact solution of the electronic Schrödinger equation to an arbitrary level of accuracy within the fixed-node approximation^31^ and has been shown to yield agreement within 0.1 kcal mol^−1^ with the quantum-chemical coupled-cluster method, which is considered the ‘gold standard' for mid-sized molecular complexes^32^. However, unlike the coupled-cluster method, DQMC scales favourably with the system size and permits calculations of larger systems^33^,^34^,^35^, including the ones of concern here. We note that relative trends in the binding energies of the complexes (Fig. 2), including the degeneracy of C1 and C2, match with the trends in the free energies of association, further supporting our focus on the binding energies.

Although DQMC provides highly accurate predictions of the binding energies, its wavefunction is not directly accessible, hindering any conceptual insight into the nature of the binding and requiring calculations at the edge of current high-performance computing. To overcome this limitation, we employ the MBD model, which is built on a quantum-mechanical Hamiltonian that can be solved exactly and at a manageable computational cost^18^,^19^, and show that the resulting wavefunctions can be interpreted in a straightforward manner. In particular, the MBD model represents each atom with a pseudoelectron in a harmonic potential, which is constructed in such a way as to reproduce the long-range dynamic response of valence electrons of an atom within a 3% accuracy^36^. The quantum charge fluctuations in such pseudoatoms are then correlated within the dipole approximation to all orders of the interaction potential, to obtain the long-range electron correlation energy^37^,^38^. The MBD method has been successfully used to model vdW interactions in a broad range of systems, ranging from small gas-phase complexes to molecular crystals^39^, to hybrid interfaces^40^, to complex nanostructured materials^34^.

Correct description of binding energetics in supramolecular complexes is challenging due to the delicate balance between different types of intermolecular interactions. Therefore, any approximate model requires a systematic verifications of its accuracy for a given class of supramolecular systems. In this regard, Fig. 2 demonstrates that MBD is fully capable of describing all three complexes C1–C3. The only deviation from the reference occurs for C3 and amounts to an underbinding of 1.5 kcal mol^−1^ (4%) outside the statistical range given by DQMC, which can be attributed to possible inaccuracies in the coupling of MBD to the underlying density functional of Perdew, Burke, and Ernzerhof (PBE) and to neglected higher-multipole coupling. To put the predictions of MBD in context, they can be compared with those of the D3 dispersion model as calculated by Antony *et al*.^24^ D3 employs a pairwise approximation to London dispersion with an optional three-body correction and, although the latter improves the bare pairwise results (see Fig. 2), the deviations from DQMC are still as much as 7 kcal mol^−1^. Without the three-body correction, the deviations would be as much as 12 kcal mol^−1^, suggesting already at this level the importance of the higher-order contributions. An explanation for why the higher-order contributions destabilize the complexes within the framework of MBD is given below.

### Changes in molecular polarizabilities

**Figure 3:**
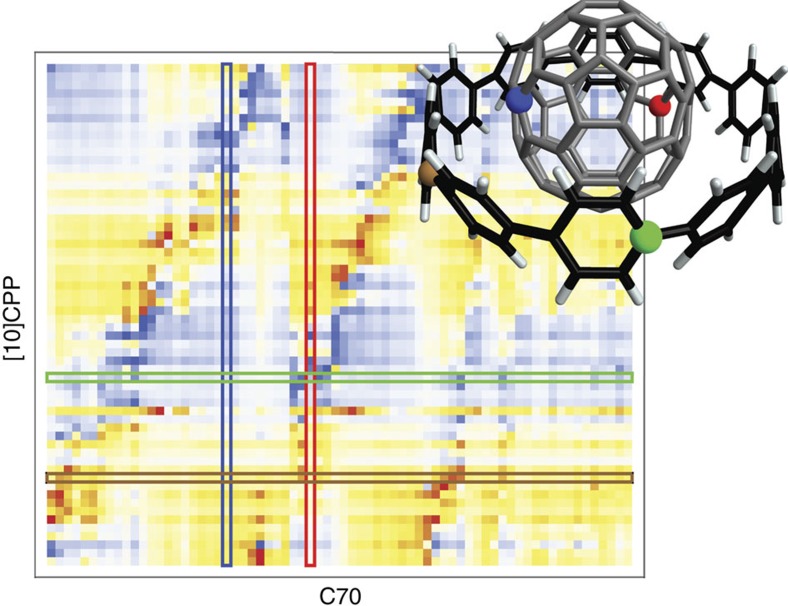
Heat map of the *xy* component of the interfragment non-local polarizability 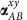 in [10]CPP–C70. Red and blue represent positive and negative values, respectively. Rows and columns correspond to carbon atoms of [10]CPP and C70, respectively, with four select carbon atoms color-coded to the marked atoms in the structure in the inset.

Having established the accuracy of MBD for the systems in question, we proceed with analysis of the mechanism of the binding by inspecting the correlated wavefunctions of the MBD Hamiltonian. The many-body correlation effects can be roughly divided into intra- and intermolecular terms. Within an isolated molecule or material, they manifest themselves in the non-trivial dependence of the total polarizability on the system size^41^,^42^. The long-range electrodynamic screening can lead to both increased or decreased polarizability with respect to sum of atomic polarizabilities, depending on the geometry and dimensionality of the system. For example, fullerenes with their relatively bulky shape are typically depolarized by these effects^18^, whereas linear and planar systems often exhibit enhanced polarizability. The latter can lead to increased stabilization of some *π*−*π* stacked systems as observed by Grimme and colleagues^13^,^43^ for linear condensed acenes. In the complexes studied here, the fullerene molecule shows 25% depolarization with respect to the sum of atomic polarizabilities, whereas the CPP rings in C1 and C2 show 31% and 35% increase, respectively. Having said that, this intramolecular portion of the electron correlation, while heavily influencing the magnitude of the intermolecular binding, is not its cause. In particular, the intramolecular correlation is captured in the diagonal blocks of the non-local polarizability **α**(**r**, **r**′), the blocks being defined by the interacting components (see Fig. 3 and Supplementary Figs 1 and 2 for plots of ***α***). The intermolecular correlation, on the other hand, is encoded in the much finer structure of the off-diagonal blocks, which is propagated into differences in the wavefunction of the whole complex with respect to those of the isolated fragments.

### Charge polarization induced by vdW interactions

**Figure 4:**
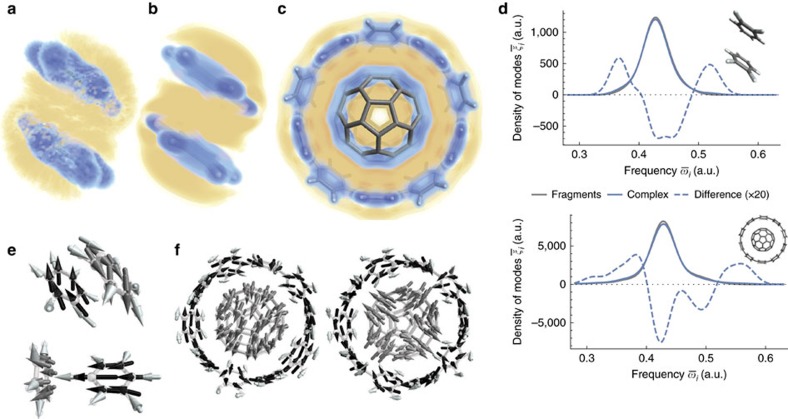
Analysis of correlated charge fluctuations in supramolecular complexes and benzene dimer. (**a**–**c**) Electron density differences (charge polarization) between a complex and its isolated fragments induced by vdW interactions. Yellow and blue colour denote accumulation and depletion of charge density, respectively. Magnitude of the density difference is mapped to saturation of the colour with 50% saturation corresponding to the density of 2 × 10^−5^ and 4 × 10^−5^ a.u. for the benzene dimer and the complex C1, respectively. Charge polarizations were obtained either from a DFT calculation with a self-consistent TS functional in the parallel-displaced benzene dimer (see Fig. 1) (**a**) or directly from the correlated MBD wavefunctions (see main text for details) for the benzene dimer (**b**) and the complex C1 (**c**). In the latter case, the relevant charge densities are calculated as expectation values of the charge density operator, 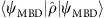. (**d**) Energy densities of the oscillation states for parallel-displaced benzene dimer (top) and complex C1 (bottom). Gaussian smoothing with half-width of 0.06 eV was applied. (**e**,**f**) Select collective fluctuation modes. The arrows represent in-phase dipole fluctuations of the electron density on the individual atoms. For benzene dimer (**e**), the lowest-energy dipole–dipole corresponds to the in-plane fluctuations on both monomers in the parallel-displaced conformation (top), but not in the T-shaped conformation (bottom). For complex C1 (**f**), the dipole (left) and quadrupole (right) in-plane oscillations are amongst the ones most contributing to the total binding energy.

In the quantum chemistry picture, the correlation of the fragment wavefunctions in conjugated complexes occurs largely via *π*→π* excitations to the unoccupied *π*-like orbitals. As these orbitals have a larger spatial extent, this correlation process leads to shift of the electrons from the atoms to the outer regions. In the density functional theory (DFT) picture, the correlation is induced by a change in the local exchange-correlation potential, which becomes decaying slightly slower with the distance from the nuclei, allowing the electrons to shift outwards. Figure 4a shows such a shift in a benzene dimer as obtained from a DFT calculation with the self-consistent Tkatchenko–Scheffler (TS) functional^44^. Finally, consider the MBD picture, where the correlation arises via coupling of the charge fluctuations within the individual fragments into collective fluctuations that may span the whole complex. These are obtained directly as single-particle solutions *φ*_*i*_ of the underlying many-body Hamiltonian 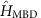 (see equation (5) in Methods),


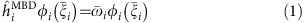


where 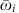 are frequencies (energies) of the coupled fluctuations, which have the form of linear combinations of the atomic fluctuations, 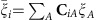. The binding combinations (in analogy to bonding orbitals) have lower energies, which leads to an increased spatial extent of the fluctuations as shown by the charge density differences in Fig. 4b. The charge densities *ρ*(**r**) are calculated explicitly from the MBD coupled wavefunctions 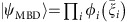 via the charge density operator 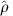 (see Methods for details),


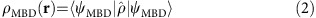


The total displaced charge (integral over charge-accumulating regions) amounts to 0.0101 and 0.0097 electrons, as obtained from the TS density functional and the MBD wavefunctions, respectively. Given the much different basis of these two approaches and the absence of any explicit parametrization of MBD with respect to spatial representation of the electron density, we find the close match between the two approaches reassuring of the solid physical foundation of the underlying charge fluctuations in MBD.

The orbital-based approaches of quantum chemistry can be linked to MBD by considering the solution of the MBD Hamiltonian as would be provided by standard quantum chemistry methods. In such an approach, the ground *s*-states of the harmonic oscillators in MBD would be correlated via virtual excitations to their first excited states, which have the symmetry of *p*-orbitals. As atomic *p*-orbitals form the basis of the molecular *π*-orbitals, this may explain why MBD is particularly fitting for description of *π*−*π* systems. (We note that this analogy is only partial though—the final states in the transitions are corresponding, but the initial states have a different symmetry.) Figure 4c illustrates that in a large supramolecular *π*−*π* complex, the charge polarization induced by vdW interactions fills the whole intermolecular region. In this case, the total displaced charge amounts to 0.11 electrons, an order of magnitude increase from the benzene dimer, which corresponds to the same increase in the vdW interaction energy.

### Formation of collective charge fluctuations

The formation of collective fluctuations in the complex is associated with a broadening of the oscillation frequencies with respect to those in the isolated fragments, which can be directly observed on the energy densities of the oscillation states. Figure 4d shows that although this broadening has a more complex shape in the complex C1 than in the benzene dimer, the overall character is similar in both cases, reflecting the fact that the mechanisms of the binding are in fact the same. We note that in contrast to the splitting of atomic orbital energies on formation of molecular orbitals, which is symmetric and facilitates the bonding via partial occupancy of the orbital space, here all states are singly occupied (the fluctuations are bosonic) and the binding arises as a result of an asymmetry in the splitting. Further insight can be obtained by analysing individual oscillation modes. Many of the collective modes have a distinct character that corresponds to global dipole, quadrupole or higher-multipole oscillations extending over the whole complex. In general, the energy ordering of the modes coincides with an increasing angular moment, with collective dipole-like fluctuations having typically the lowest energy. However, this is not always the case as can be observed on the two nearly degenerate conformations of the benzene dimer. Figure 4e shows that in the parallel-displaced conformer (top), the lowest-energy collective fluctuation corresponds to two aligned in-plane dipole fluctuations in the monomers, whereas in the T-shaped conformer (bottom) the corresponding collective mode comprises one in-plane and one out-of-plane monomer fluctuation, the latter having a substantially lower polarizability. These observations are manifested in the vdW interaction energies as well, which are reduced by the higher-order many-body effects by 8% and 3% in the parallel-displaced and T-shaped conformation, respectively. In the supramolecular complex C1 (Fig. 4f), the fluctuations most contributing to the binding correspond to dipole and quadrupole oscillations spanning the whole complex. This can be related to the standard London picture of pairwise dispersion, where fluctuating dipoles on individual atoms are being correlated. Figure 4f demonstrates that this view can be generalized to the case of large complexes not via sum over the atom pairs, but rather by considering collective fluctuating dipoles and higher multipoles of the whole molecules, akin to wavelike dipole fluctuations or plasmons in nanomaterials^45^. In this framework, the destabilization by the many-body correlation effects with respect to the second-order (pairwise) approximation can be understood by considering the degrees of freedom of the correlation. In the pairwise picture, the oscillations within each atom pair are correlated independently, essentially resulting in an ‘overcorrelation'. In contrast, the collective fluctuation model correlates all atomic dipole oscillations at once, allowing only for a moderate amount of correlation. As the differences in the degrees of freedom grow with the system size, the many-body effects are more pronounced in the supramolecular complexes, where they reduce the binding energies by as much as 16%, or 6 kcal mol^−1^, in the case of the buckyball catcher.

### Trends across structural motifs

**Figure 5:**
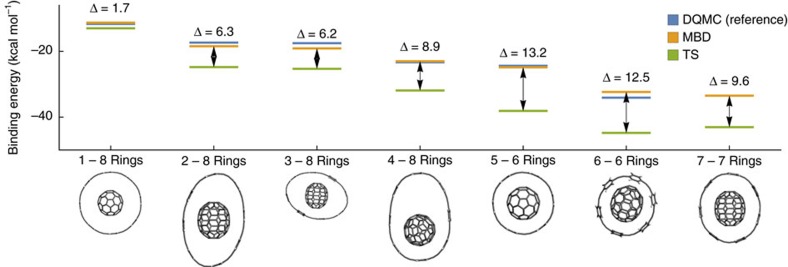
Interaction energies of C70 in [*N*]cycloparaphenyleneacetylene (6≤*N*≤8). The sample was selected to cover a broad spectrum of geometrical motifs and hence the individual geometries are not necessarily energy minima. However, all geometries are stationary points of the potential energy surface. The energies are calculated with respect to relaxed fragments (see Supplementary Data 1). DQMC is the diffusion quantum Monte-Carlo method (not given for system 7), whereas MBD and TS are the MBD model and TS method calculated on top of the PBE functional, respectively. Δ denotes the energy difference between MBD and TS in kcal mol^−1^. Numerical values are presented in Supplementary Table 2.

We have demonstrated how collective charge fluctuations are responsible for binding in the three studied supramolecular complexes, and that quantitative models, which take this into account, provide accurate predictions of the binding energies. The last part of our work deals with two questions: (i) are these findings transferable to other conjugated systems? (ii) Are they characteristic of conjugated systems? The magnitude of the binding and especially the contribution of the higher-order many-body correlation effects is a combined result of several factors including the degree of symmetry of a system, its topology, compactness, and mutual orientation of the host and the guest. To investigate whether our model can account for these different factors, we have calculated the binding energies of a set of conformations of the C70 fullerene hosted in [*N*]cycloparaphenyleneacetylene rings, with *N* ranging from 6 to 8 (see Fig. 5). The geometries were selected to cover a spectrum of interaction motifs, ranging from tightly stacked to open structures, and containing both symmetric as well as distorted cases. The case of *N*=6 was previously studied by Yuan *et al*.^46^ but their focus was mostly on equilibrium structures. The MBD binding energies are compared with the results of DQMC as well as the TS method^36^, which is a reliable model for vdW interactions in smaller systems and simple solids, but neglects the higher-order correlation effects. As in the case of the CPP-based complexes, MBD provides binding energies in excellent agreement with DQMC, with the largest difference of 1.7 kcal mol^−1^ (5%) and the mean absolute difference of 0.9 kcal mol^−1^. Furthermore, there is no clear pattern in the remaining small differences, suggesting that MBD treats the various types of geometries included in the sample on an equal footing. In contrast, the pairwise TS method in general overbinds, with differences ranging from 2 to 13 kcal/mol. Part of this overbinding can be attributed to the lack of short-range screening in TS; however, most of the specific differences on individual systems stem from the ‘overcorrelation' introduced above. We observe that these differences are largest in the tightly stacked structures, resulting in the largest overbinding and a seeming stability of these systems. With MBD and DQMC, on the other hand, these structures are much closer in binding energy to the more extended conformations.

### Analysis of individual fluctuation modes

**Figure 6:**
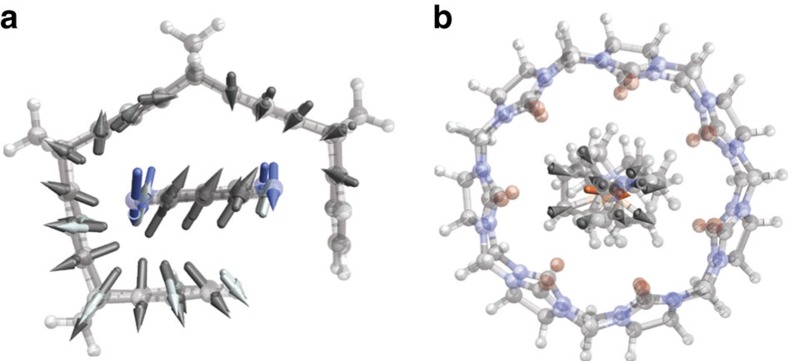
The most binding fluctuation mode in two supramolecular complexes. Only atoms where the magnitude of the dipole oscillation is larger than 5% of the largest oscillation are shown. (**a**) Conjugated ‘tweezer' complex. (**b**) Electrostatically bound cucurbituril complex.

vdW interactions in all materials are caused by correlation of charge fluctuations and we have shown above that these fluctuations are non-local and collective in the case of conjugated *π*−*π* systems. To investigate whether such characteristics are specific to these systems, we have analysed the fluctuation mode that contributes most to the total binding energy in seven select complexes from the data sets S12L^23^ and S8 (ref. 24; see Supplementary Figs 3 and 4), covering all distinct types of bonding therein, which range from *π*−*π* stacking (two ‘tweezer' complexes, one ‘pincer' complex and the [10]CPP complex studied above) to hydrogen bonding, to electrostatic interactions (a pseudorotaxane complex, an amide macrocycle complex and a cucurbituril complex). The fluctuation modes of a complex can be expanded in terms of the fluctuation modes of the individual monomers with equation (10) (see Supplementary Methods for details). A simple yet revealing measure of the number of components of such an expansion is the inverse of the largest expansion coefficient (as an example, consider the expansion 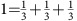, where this measure would be 3). In the four conjugated supramolecular complexes, we find this measure to be 2.3, 2.8, 3.0 and 4.4, whereas in the three complexes with other types of bonding, we find 1.1, 1.1 and 1.7, demonstrating that the modes in the former group are significantly more collective. As an illustrative example, the most contributing fluctuation mode in the *π*−*π* stacked ‘tweezer' complex in Fig. 6a corresponds to a collective dipole-like fluctuation, whereas it is mostly localized and unordered on the guest molecule in the electrostatically bound cucurbituril complex in Fig. 6b. In the latter case, the mode is strongly binding because of its large polarizability and unspecific correlation with the modes of the host molecule. Although performed on a limited number of systems, these preliminary results already suggest that the collective charge fluctuations indeed are characteristic of the conjugated complexes.

## Discussion

In previous works, collective charge fluctuations (molecular plasmons^47^) have been discussed predominantly within the context of solids and low-dimensional extended systems^15^,^16^,^17^. Our findings show that they serve as a natural description of long-range electron correlation in molecular systems, not only at the asymptotic limit, but at equilibrium distances as well, and furthermore that the corresponding models can be made fully quantitative. We have demonstrated that using a relatively simple Hamiltonian model for these fluctuations, one can: (i) calculate binding energies of supramolecular complexes within chemical accuracy with respect to high-level reference data, (ii) obtain both quantitatively and spatially correct charge polarization due to long-range electron correlation with respect to density-functional calculations, and (iii) analyse and visualize the individual fluctuations and their contributions to the total binding energy.

Two different models (TS and D3), which do not account for the collective charge oscillations, are not capable of predicting binding energies of a comparable accuracy. Our analysis also suggests that such fluctuations are especially important in conjugated systems in comparison with complexes with other types of bonding. One of the main characteristics of aromatic systems is their relatively narrow HOMO-LUMO gap, making them an interesting point on the zero-gap to large-gap spectrum^17^,^48^. An essential property of the dipole oscillators used in our model is that they are not point dipoles, but rather have a natural width. This is crucial for predicting accurate molecular polarizabilities, but also seems to effectively provide the amount of delocalization in the molecular response that is required to describe long-range electron correlation in finite-gap systems such as those studied in this work. The fully delocalized electrons that can be found in metallic systems and that can lead to type-C non-additivity as classified by Dobson^49^ are not explicitly accounted for by our model and this challenge is currently the topic of our work.

## Methods

### Many-body dispersion

MBD^18^,^19^,^50^ models the electrons of each atom as a harmonic oscillator with the static polarizability *α*_0_ and oscillation frequency 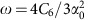 calculated from the *C*_6_ dispersion coefficient. These parameters are obtained from the electron density by scaling the corresponding free-atom values with a ratio of the Hirshfeld volumes of the atoms in the molecule and free atoms,


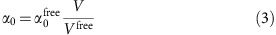


The scaled atomic polarizabilities are then screened via the Dyson screening equation using the short-range part of the dipole potential.


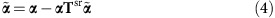


The resulting fully non-local polarizability 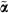 (see Supplementary Fig. 1) is then contracted back to individual atoms. The polarizabilities obtained in this way are then used as an input into the MBD Hamiltonian,


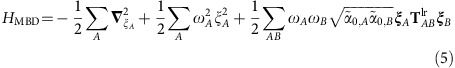


where 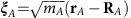 is the mass-weighted displacement of the pseudoelectron on atom *A* from its equilibrium position **R**_*A*_, and **T**^lr^ is the long-range part of the dipole potential. The solution is obtained by direct diagonalization which is possible due to the biquadratic form of the Hamiltonian and leads to the set of coupled fluctuation frequencies 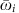 and harmonic modes 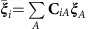. The MBD energy is then given by a plasmon-pole formula


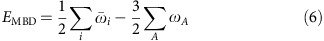


An alternative and fully equivalent formulation, which allows for expansion of the energy in orders of the coupling, starting with the second order, is provided by the random phase approximation,


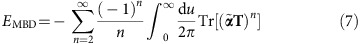


The MBD charge densities are obtained from the correlated wavefunctions


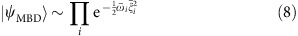


as an expectation value of the charge density operator


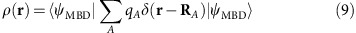


where the charges are set to 1 and the effective masses of the pseudoelectrons then follow from *α*_0_=*q*/*mω*^2^. The collective fluctuation modes 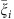 of the complex can be expressed in terms of the modes 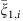, 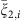 of its fragments via


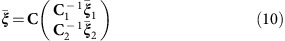


All MBD calculations were performed with a standalone program available in ref. 51.

### Diffusion quantum Monte Carlo

All DQMC energies presented in this work were calculated using the CASINO programme^52^, employing relativistic pseudopotentials^53^ with the locality approximation^54^ and the Slater–Jastrow trial wave functions


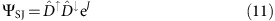


where 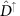, 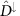 are Slater determinants constructed from single-particle spin orbitals representing the up and down electron-spin projections, respectively, and e^*J*^ is the so-called Jastrow factor, which is an exponential of a sum of explicitly correlated one- (electron–nucleus), two- (electron–electron) and three-body (electron–electron–nucleus) terms. Single-particle orbitals were obtained from local density approximation (LDA) plane-wave calculations performed with the PWSCF program^55^ and expanded in terms of B-splines^56^. DQMC binding energies have been computed with respect to the fragments separated by at least 10 Å, with the residual binding energy of less than 0.6 kcal mol^−1^ (estimated with MBD). This procedure avoids the size-consistency problems of DQMC (Zen, A. *et al*., in preparation).

### DFT calculations

All DFT calculations were performed with the FHI-aims program^57^ using the ‘tight' settings for basis sets and real-space grids. FHI-aims is an all-electron code with numerical basis sets. The Kohn–Sham self-consistent cycle was converged to 10^−6^ eV in energy and 10^−5^ electrons in density.

### Data availability

The authors declare that the data necessary for reproducing the results of this study are available within the article and its Supplementary Information files.

## Additional information

**How to cite this article:** Hermann, J. *et al*. Nanoscale *π*–*π* stacked molecules are bound by collective charge fluctuations. *Nat. Commun.* **8,** 14052 doi: 10.1038/ncomms14052 (2017).

**Publisher's note**: Springer Nature remains neutral with regard to jurisdictional claims in published maps and institutional affiliations.

## Supplementary Material

Supplementary Information Supplementary figures, supplementary tables and supplementary methods.

Supplementary Dataset 1 XYZ geometry files for all molecules studied in the manuscript.
