# Supplementary material for: Nanoscale π–π stacked molecules are bound by collective charge fluctuations
Source: Nat Commun. 2017 Feb 7;8:14052. doi: 10.1038/ncomms14052 (PMC5309697; doi:10.1038/ncomms14052)
Supplement: Supplementary Information — Supplementary figures, supplementary tables and supplementary methods. [file ncomms14052-s1.pdf]

Supplementary Information

Supplementary Methods

The contribution of the individual fluctuation modes of the complex to the total binding energy is given by

$$\mathbf{P}^2\big((\mathbf{P}^\mathrm{T})^2\boldsymbol{\omega}_\text{complex}-\boldsymbol{\omega}_\text{fragments}\big),\qquad \mathbf{P}=\mathbf{C}^\mathrm{T}\begin{pmatrix}\mathbf{C}_1 & \mathbf{0}\\ \mathbf{0} & \mathbf{C}_2\end{pmatrix}\tag{SE 1}$$

where $\mathbf{P}$ is the projector from the fragment to the complex modes, $\mathbf{C}$ and $\mathbf{C}_k$ are columnwise matrices of the modes of the complex and the fragments, respectively, and $\boldsymbol{\omega}_S$ is a vector of the oscillation frequencies of system S. This projection ensures that almost no complex modes are anti-binding. Supplementary Figs. 3 and 4 contain the analysis of the most binding fluctuation mode in seven supramolecular complexes. In conjugated complexes, this mode is always delocalized over the whole system and is composed of several monomer modes. In non-conjugated complexes, on the other hand, it is much more localized and dominated by a single monomer mode.

Supplementary Tables

Supplementary Table 1 | Interaction energies (kcal/mol) of the complexes C1–C3.

method	n-body	C1	C2	C3
DQMC (reference)	all (electrons)	−41.2 ± 1.0	−41.5 ± 1.0	−30.1 ± 0.8
MBD	all (dipoles)	−40.6	−39.0	−29.6
TS	2	−60.9	−55.2	−45.5
DFT-D3 w/ 3-body	2+3	−48.4	−41.9	−32.6
DFT-D3	2	−52.9	−45.8	−36.1
PBE	–	13.3	3.7	12.4

Supplementary Table 2 | Interaction energies (kcal/mol) of the cycle complexes. [a]The row order corresponds to the x-axis order in Supplementary Fig. 4 of the main text.

complex[a]	MBD	TS	PBE	DQMC
[8]CPPA–C70 “circle”	−11.3	−13.0	−1.1	−11.7
[8]CPPA–C70 “lying along”	−18.5	−24.8	8.0	−17.4
[8]CPPA–C70 “lying cross”	−19.1	−25.3	5.9	−17.5
[8]CPPA–C70 “upright side”	−23.0	−31.9	8.9	−23.3
[6]CPPA–C70 “upright in”	−24.9	−38.1	30.6	−24.4
[6]CPPA–C70 “tilted out”	−32.4	−44.8	11.2	−34.1
[7]CPPA–C70 “lying in”	−33.5	−43.1	1.5	–

Supplementary Figures

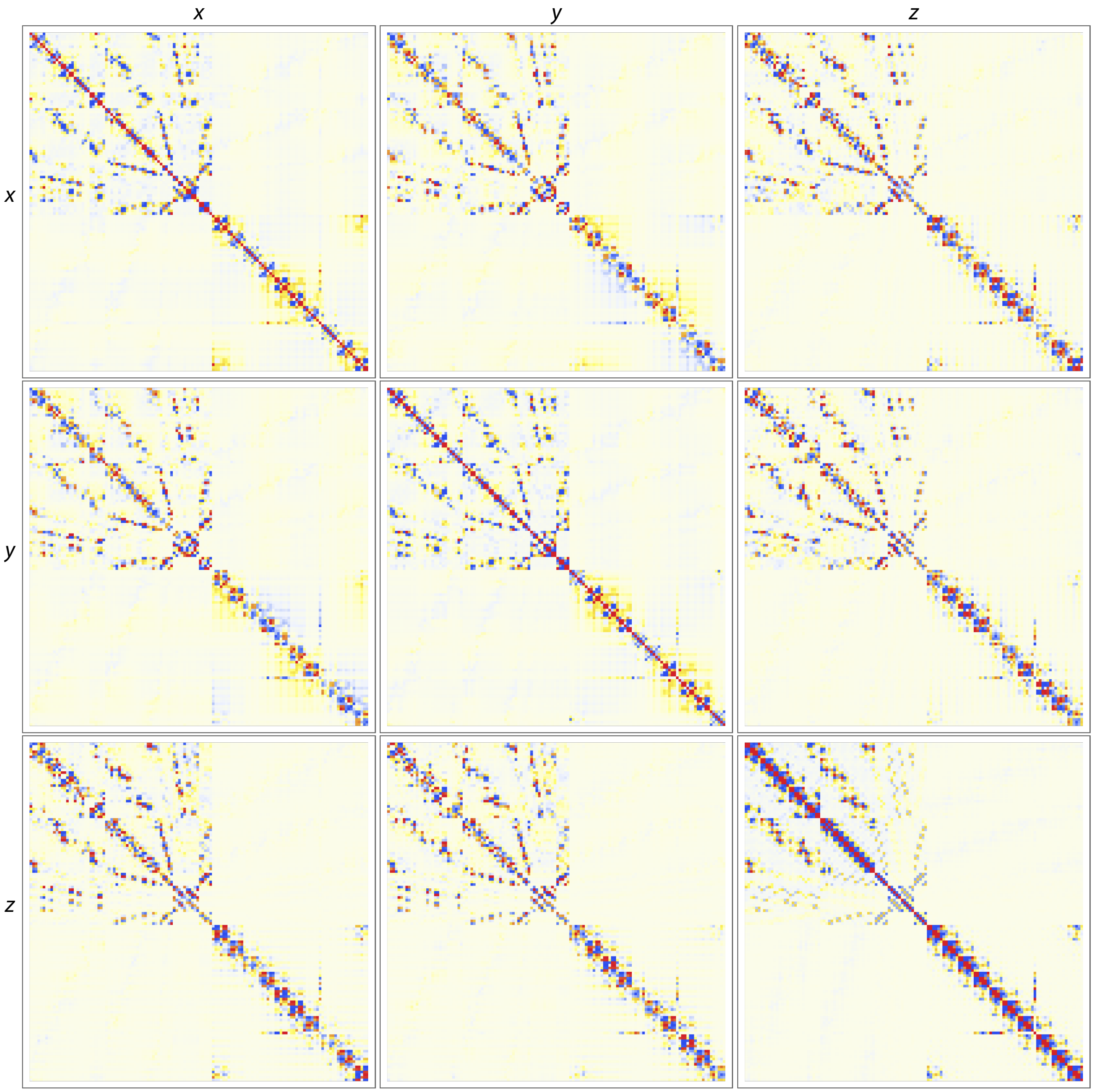

Supplementary Figure 1 | Heat map of the non-local polarizability α_{AB} in [10]CPP–C70. Red and blue represent positive and negative values, respectively. Cartesian "channels" are displayed in separate matrices, atoms correspond to rows and columns. First 70 atoms form the fullerene C70, the rest comprises [10]CPP. Only carbon atoms are considered.

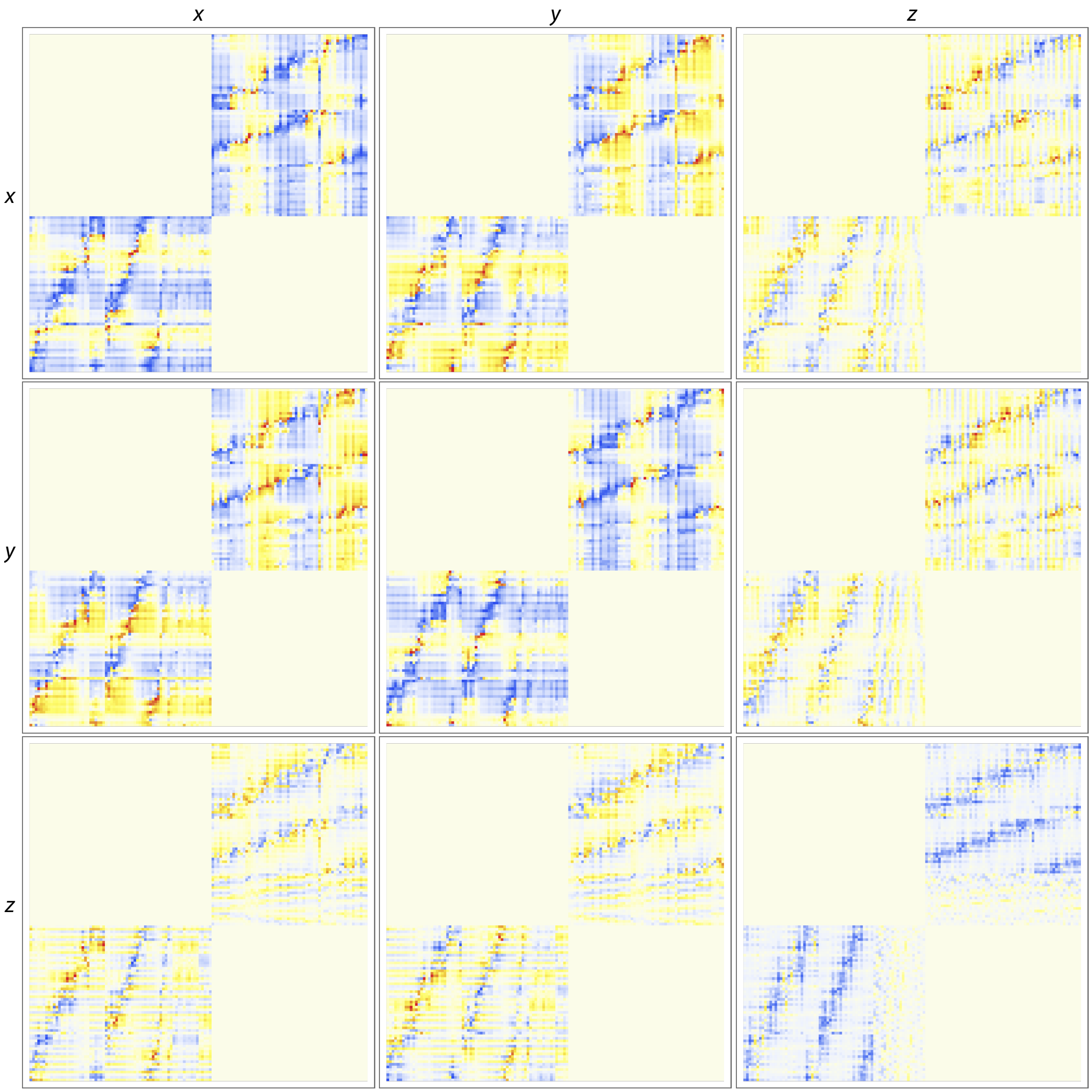

Supplementary Figure 2 | Heat map of the interacting part of non-local polarizability α_{AB} in [10]CPP–C70. The representation is the same as in Supplementary Fig. 1, but the intramolecular matrix elements have been blanked and the intermolecular elements amplified.

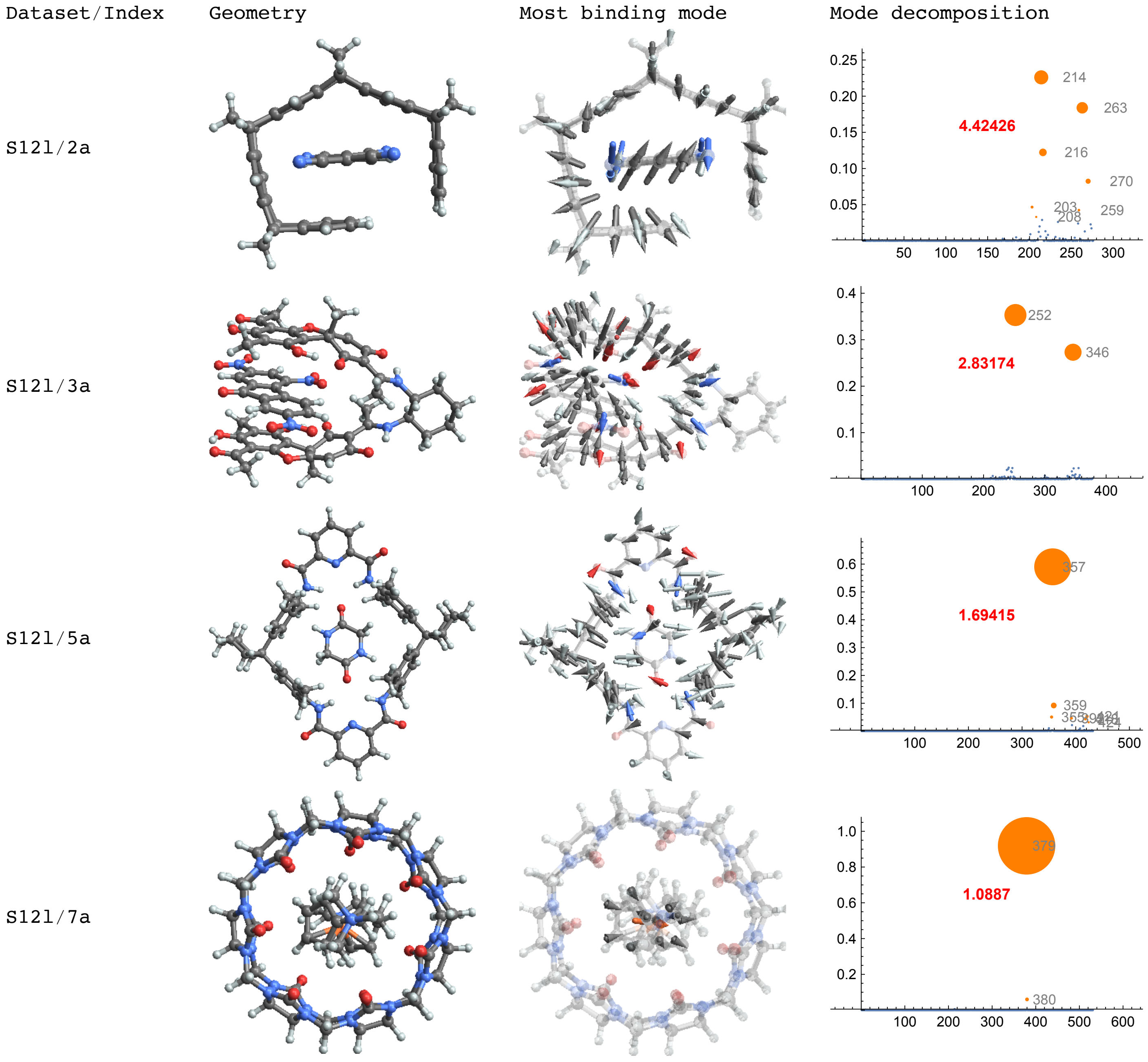

Supplementary Figure 3 | Decomposition of the most binding fluctuation mode for select complexes from the S12L benchmark dataset. The right-most column contains plots of the squares of the coefficients of the expansion of most binding complex mode into the monomer modes. The number in red is the inverse of the largest expansion coefficient. As such, it is a simple measure of how many monomer modes is the complex mode composed of.

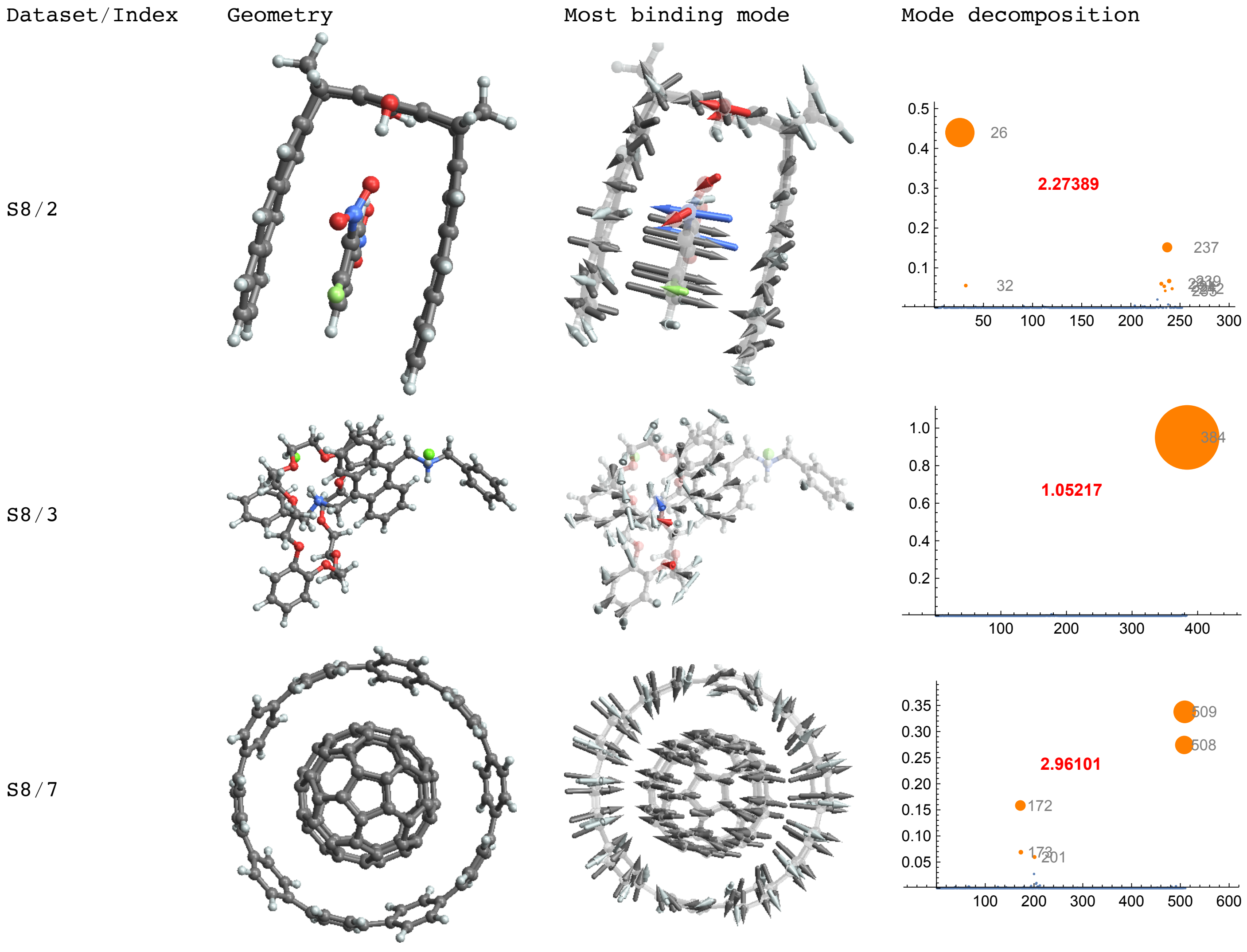

Supplementary Figure 4 | Decomposition of the most binding fluctuation mode for select complexes from the S8 benchmark dataset. See the caption of Supplementary Fig. 3 for more details.
